# Supplementary material for: Vitamin E Ameliorates Impaired Ovarian Development, Oxidative Stress, and Disrupted Lipid Metabolism in Oreochromis niloticus Fed with a Diet Containing Olive Oil Instead of Fish Oil
Source: Antioxidants (Basel). 2023 Jul 29;12(8):1524. doi: 10.3390/antiox12081524 (PMC10451663; doi:10.3390/antiox12081524)
Supplement: Supplementary file 1 [file antioxidants-12-01524-s001.zip › antioxidants-2489865-supplementary.pdf]

Table S1 Primer sequences used for qRT-PCR

Primer	Primer sequence (5'-3')	NCBI GenBank accession no
erα-F	AGCGACAAATCAGTGCACCA	NM_001279770.1
erα-R	ATTGCACGCTTCCTTCCATC	
erβ-F	AGCATCCAAGGACACAACGA	NM_001279774.1
erβ-R	ATGCCCTTTGGTTCAGCTGT	
fshr-F	ACGCCTGCATCATCATGACA	NM_001279588.1
fshr-R	GCTGATGAAGGCTTGCGAAA	
lhr-F	ACAAGCTGACAGTACCTCGC	XM_005474002.4
lhr-R	CTGACAGCTCCCCACCAAAA	
pparα-F	TCCAAAAGAAGAACCGAAACA	NM_001290066.1
pparα-R	TTCCACCTCTTTCTCAACCAT	
pparγ-F	TTTACCCATCAAACTGACCAC	NM_001290200.1
pparγ-R	GAGGAAATGGAGGCGTAGT	
hsl-F	CGAAAGTTTTGTCCGTGAATA	FJ601660.1
hsl-R	CTGATGCCGCTTGTAGTTTTC	
accα-F	GCGGTGTTCGGCTTGTTTTT	XM_025910662.1
accα-R	CAAGTCCACCTTCCCTTGGT	
elovl6-F	ACAGTTCAACGAGGACGAAGC	XM_003443399.5
elovl6-R	AGCAAGGGTGAGTGACCACAG	
nrf2-F	CTGCCGTAAACGCAAGATGG	XM_003447296.5
nrf2-R	ATCCGTTGACTGCTGAAGGG	
keap1-F	CTTCGCCATCATGAACGAGC	XM_003447926.4
keap1-R	CACCAACTCCATACCGCACT	
nf-κb-F	GCAGAAGGAGGCAGTGGAAG	XM_019363515.2
nf-κb-R	GACCTGCTGTGTTGGTTTGGT	
tnfα-F	GGAAGCAGCTCCACTCTGATGA	JF957373.1
tnfα-R	CACAGCGTGTCTCCTTCGTTCA	
infγ-F	AAGAATCGCAGCTCTGCACCAT	FJ601660.1
infγ-R	GTGTCGTATTGCTGTGGCTTCC	
il1β-F	CAAGGATGACGACAAGCCAACC	XM_019365843.2
il1β-R	AGCGGACAGACATGAGAGTGC	
ef1α-F	ATCAAGAAGATCGGCTACAACCCT	NM_001279647.1
ef1α-R	ATCCCTTGAACCAGCTCATCTTGT	

β-actin-F	CCACACAGTGCCCATCTACGA	EU887951.1
β-actin-R	CCACGCTCTGTCAGGATCTTCA	

Table S2 Fatty acid composition in the ovary tissues (% of total fatty acids)

Fatty acid	70 OO	200 OO	70 FO	200 FO	two-way ANOVA		
					Lipid	Ve	Interaction
C12:0	0.18±0.04	0.15±0.01A	0.25±0.05	0.21±0.01B	ns	ns	ns
C14:0	2.66±0.16	2.52±0.08	3.04±0.11	2.62±0.15	ns	ns	ns
C15:0	0.13±0.02	0.15±0.01A	0.23±0.03	0.20±0.01B	**	ns	ns
C16:0	28.91±0.64	29.64±0.13	30.81±0.53	30.25±0.33	*	ns	ns
C17:0	0.22±0.03	0.23±0.01A	0.35±0.05	0.31±0.01B	**	ns	ns
C18:0	17.25±0.16A	16.31±0.58A	23.29±0.29B	23.39±0.23B	**	ns	ns
C20:0	0.31±0.04	0.39±0.04	0.50±0.06	0.49±0.02	**	ns	ns
C22:0	0.23±0.06	0.27±0.02A	0.47±0.11	0.42±0.02B	*	ns	ns
C16:1	1.95±0.17	1.75±0.06A	2.30±0.20	2.20±0.05B	*	ns	ns
C18:1	38.40±0.84A	37.21±0.36A	25.24±0.65B	23.55±0.11B	**	*	ns
C20:1	1.28±0.11	1.30±0.05	1.36±0.03a	1.23±0.01b	ns	ns	ns
C22:1	0.22±0.05	0.25±0.02A	0.45±0.08	0.38±0.02B	**	ns	ns
C18:2n-6	2.98±0.09aA	3.78±0.03b	1.57±0.19aB	3.01±0.16b	**	**	ns
C18:3n-3	0.17±0.02	0.20±0.03	0.13±0.02	0.22±0.02	ns	ns	ns
C18:3n-6	0.27±0.04A	0.27±0.03A	0.11±0.02B	0.14±0.01B	**	ns	ns
C20:2n-6	0.32±0.05	0.39±0.03A	0.27±0.01	0.28±0.01B	*	ns	ns
C20:3n-6	0.69±0.04	0.66±0.05	0.63±0.03a	0.72±0.01b	ns	ns	ns
C20:3n-3	0.07±0.01	0.09±0.01	0.10±0.02	0.11±0.01	ns	ns	ns
C20:4n-6	1.09±0.02aA	1.31±0.01bA	2.51±0.08aB	2.97±0.05bB	**	**	*
C20:5n-3	0.21±0.01A	0.29±0.03A	1.05±0.07B	1.31±0.11B	**	*	ns
C22:3	0.07±0.02A	0.10±0.01A	0.17±0.03aB	0.40±0.05bB	**	**	*
C22:4n-6	0.19±0.06	0.25±0.02A	0.40±0.05	0.39±0.01B	**	ns	ns
C22:5n-3	0.28±0.08	0.33±0.03A	0.71±0.18	0.65±0.04B	**	ns	ns
C22:6n-3	1.85±0.04aA	2.15±0.08bA	4.07±0.03aB	4.55±0.10bB	**	**	ns
Σ SFA	49.90±0.72A	49.67±0.44A	58.95±0.38B	57.89±0.33B	**	ns	ns
Σ MUFA	41.85±0.90A	40.50±0.43A	29.34±0.62aB	27.35±0.08bB	**	*	ns
Σ PUFA	8.17±0.37aA	9.82±0.31bA	11.71±0.41aB	14.75±0.30bB	**	**	ns
Σ n-3 PUFA	2.58±0.16A	3.07±0.16A	6.06±0.21aB	6.84±0.18bB	**	**	ns
Σ n-6 PUFA	5.53±0.20a	6.66±0.14b	5.48±0.30a	7.51±0.41b	ns	**	ns

Notes： means with different lowercases in the same dietary lipid with different V_E level are significantly different from each other; means with different largercases in the same dietary V_E level with different lipid source are significantly different from each other（**：$P < 0.01$；*：$P < 0.05$；ns：$P > 0.05$).
